# Psycho-social outcome in liver transplanted children: beware of emotional self-assessment!

**DOI:** 10.1186/1824-7288-38-37

**Published:** 2012-08-10

**Authors:** Ana Maria Calinescu, Valérie A McLin, Dominque Belli, Barbara E Wildhaber

**Affiliations:** 1 Division of Pediatric Surgery, Department of Pediatrics, University Children’s Hospital of Geneva, Geneva, Switzerland; 2 Unit of pediatric liver transplantation and hepatology, Department of Pediatrics, University Children’s Hospital of Geneva, Geneva, Switzerland; 3 Service de Chirurgie Pédiatrique, Département de l’enfant et de l’adolescent, Rue Willy Donzé 6, 1211, Genève / Suisse, Switzerland; 4 Unité de Gastroentérologie et Hépatologie pédiatrique, Département de l’enfant et de l’adolescent, Rue Willy Donzé 6, 1211, Genève / Suisse, Switzerland

**Keywords:** Psycho-social outcome, Pediatric liver transplantation, Quality of life

## Abstract

**Background:**

Psycho-social outcome in children after liver transplantation (LT) is known to be inferior to age-related peers. Yet, when children and their parents are questioned by their nurse or physician about the child’s psycho-social well-being, the answers usually are very positive. We hypothesized that patients and their parents after LT report their psycho-social well-being too enthusiastically when enquired by their personal care takers.

**Methods:**

Inclusion criteria: LT at the Children’s University Hospital of Geneva 1992–2007, age >3 years, <16 years, time after LT >2 years. Children and their parents were questioned by their well-known, familiar nurse at the annual follow up visit about their personal well-being. To allow for evaluation of answers, scores (good, medium, bad) were attributed to the different questions. 46 children were included in the study.

**Results:**

Mean age at enquiry was 9.7 years (SD 4 years), mean time after LT was 7.5 years (SD 4.2 years). The different themes were reported as good for: parent–child relationship (83%), relationship with peers (98%), relation with siblings (39%), sport activities (54%), play activities (78%), school performance (87%), expression skills (67%), and general behavior (89%).

**Conclusion:**

Most of our LT children and their parents consider, during a personal interview with a closely related, familiar nurse, that the child’s psycho-social outcome is good. Yet, it is generally acknowledged that children after LT have negatively altered psycho-social outcomes. Thus, emotionally influenced reports about psycho-social outcome in children after LT must be looked at with care.

## Introduction

When evaluating the success of our liver transplantation (LT) program, we mainly used to take into account the patient’s survival (90%) and graft survival (82%), our hospital being the only Swiss centre performing LT in children [1]. Yet, nowadays more and more attention is paid to the assessment of the health related quality of life (HRQOL) of our pediatric liver transplanted patients [2,3]. In the near future outcomes in LT might even be judged by the quality of life of the years restored by LT, a measure that might comprise both quality of life and survival rates [4].

The quantification of HRQOL in these patients remains a matter of debate. There is no “gold standard” that might measure the different concerns specific to this patient population [2,3]. Further, HRQOL needs to be assessed from several points of view: physical health, mental health, social functioning, role functioning and general health perception [5]. Investigators that previously examined this subject have clearly shown a lower HRQOL in liver transplanted children reported to normal healthy patients and equal to patients with chronic illness [6-9]. This is of most importance, since children with lower HRQOL will show impaired compliance and lower adherence to medical treatment, and thus may present with worse clinical outcome than those who are happy and psycho-socially stable [10-14].

The present study was conducted to challenge the psycho-social outcome of our Swiss pediatric liver transplantation population. We aimed to identify whether the perception of the patient’s well being, when inquired by the medical staff at the annual follow-up visit, is correct. Taking into account that, in the past, their answers usually had been very positive, we hypothesized that liver transplanted children and their parents report their psycho-social well being (too) enthusiastically when questioned by their personal, familiar medical care-takers.

## Methods

### Study population

Patients aged 3 to 16 years who underwent LT between 1992 and 2007 at the University Children’s Hospital of Geneva, with a survival of more than two years after LT and receiving a follow-up care in University Children’s Hospital of Geneva, were included in the study. A minimum of two years after LT was thought to be appropriate, since it has been shown that psycho-social outcomes significantly improve in the first six months after LT and remain rather stable thereafter [5]. Upon review of our local LT database we identified 46 children, all of them being included in the final study population. The study was approved by the Ethical Committee of the University Hospitals of Geneva.

### Study design

The present qualitative study is a single centre cross sectional study. We used a semi-structured interview (Table 1), conducted by the transplant team nurse at the annual follow up visit. One of the or both parents together with the child were questioned by their well-known, familiar nurse before the clinical assessment. The order of asking the questions was random. The time for the interview was 30 to 40 minutes. A semi-structured questionnaire was used in order to allow flexibility for the nurse, and to let new questions to be brought up during the interview, as a result of what the answers were, but still giving a framework of themes to be explored.

**Table 1:** Questionnaire used in the semi-structured interview

A	Social situation
	How is the relationship with parents, siblings and peers?
	How could you describe your playing activities?
B	Illness / liver transplantation
	Medical condition?
	Are you physically active?
	Do you think that the liver transplantation limited your physical activities?
	Are you satisfied with your body?
C	Education
	Have you finished primary school, high school?
	Do you have school retardation?
	Do you have career plans?
D	Psychological status
	How would you describe your general behaviour?
	Do you think that you are expressing yourself properly?

### Analysis

Scores (good, medium and bad) were attributed to the different questions from our questionnaire to allow for evaluation of the answers. For analysis, the statistical programme SPSS 18 was used.

## Results

Of the 46 children 27 (59%) were transplanted for biliary atresia, 13 (28%) for other cholestatic liver disease, 4 (9%) for metabolic disease and 2 (4%) for fulminant hepatitis. Mean age at inquiry was 9.7 years (standard deviation 4 years), mean time after LT was 7.5 years (standard deviation 4.2 years).

**Figure 1:**
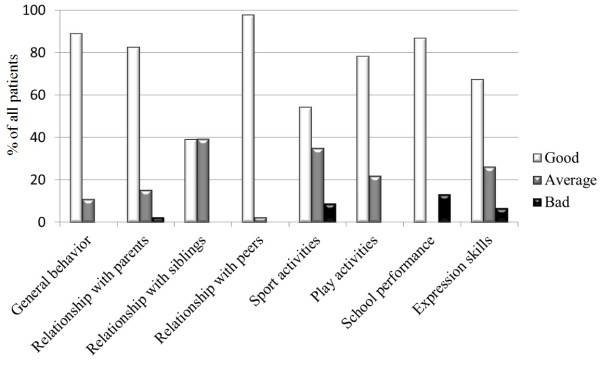
Most of our LT children and their parents consider, during a personal semi-structured interview with a closely related, familiar nurse, that the child’s psycho-social outcome is good.

The parent child relationship was described as good in 38 cases (82.6%), medium (“tense”) in 7 (15.2%) cases, and bad in 1 case (2.2%). The relationship with peers was seen as good in 45 (97.8%) children, as medium (“conflictual”) in 1 case (2.2%), and no child described a bad relationship with peers. The relationship with siblings was considered as good in 18 (39.1%) children and as medium for 10 (39.1%) children. Of note, 10 children and parents didn’t want to or couldn’t express their opinion on this topic. The physical stamina when performing sports was described as good in 25 (54.3%) children, as medium in 16 (34.8%) and bad for 5 patients (10.9%). Play activities was thought to be good in 36 (78.3%) patients, in 10 (21.7%) as medium, no patient/parent described a bad playing activity. Regarding educational data, the school activity was perceived as being good in 40 (87%) patients and bad in 6 (13%). Of note, this item was measured in years of school retardation: if no retardation was mentioned it was considered as good; at the presence of retardation it was classified as bad. The social expressional skills were considered as good in 31 (67.4%) patients, as medium in 12 (26.1%) and as bad in 3 patients (6.5%). The patient’s general behavior was seen as good in 41 (89.1%) patients and as medium in 5 (10.9%). No patient or parent described a bad behavior. Results are graphically summarized in Figure 1.

## Discussion

It is generally accepted that outcomes after LT nowadays represent more than just survival rates: a child after LT does not only need physical follow-up, but also psycho-social evaluation [4]. In order to have a rigorous approach to the health status of LT patients physicians must examine not only physical but also the psycho-social outcomes [7]. Cross sectional studies show, for example, that psycho-social outcome in LT children is inferior to healthy peers and equal to children receiving cancer therapy [4]. Indeed, a LT child is not a cured, healthy child; it is a child for whom a fatal disease was substituted by a persistent, controlled, “ill-like” condition, which has associated morbidities that might be stable or evolve despite a well preserved allograft function [2,3]. Thus, children and their parents must face the burden of ongoing medical care and the anxiety about the child’s future health [8]. In this particular context, the importance of evaluating the psycho-social outcomes is given: 1) by the fact that it is a way of hearing the child’s own voice and perspective about his illness; 2) the measure of psycho-social outcomes might predict the future health and mortality of our patients [15]; and 3) an inferior psycho-social adjustment might be a cause as well as a result of non-adherence and non-compliance [14]. The non-adherence rate in pediatric LT recipients is about 30% and might contribute to 15% of graft loss [16]. Further, psycho-social evaluation may identify patients at risk for poor psycho-social long-term outcomes and allow us to offer anticipatory guidance and targeted interventions [4].

The measurement of HRQOL in LT children, and of their psycho-social outcome in particular, is controversial, as there is no “gold standard” instrument [2,3]. The tools we use must be age-specific and sensitive to developmental changes [16]. To date the instruments used are generic, allowing applicability across many types of disease, treatments and types of individuals. The use of disease specific tools would allow us to detect aspects relevant to LT children. For the younger age groups researchers only rely on parental assessment [9], but data must, whenever possible, be directly obtained from children, given the differences noticed between the child and parent reports [2,3]. The psycho-social assessment should be performed ideally by a mental health provider, familiar with the transplant scenario [11].

When our LT patients and their parents were inquired about their psycho-social outcomes by their well-known nurse at the annual follow up visit, their answers were very good in a striking majority. Knowing the latest literature in the field, our study must be considered as inaccurate to judge the real psycho-social status of our LT children. Our data suggest that emotionally influenced reports about psycho-social outcomes in children after LT must be interpreted with greatest care.

What are the reasons for the failure of this well-intended study? Children and parents may be excessively enthusiastic about their psycho-social well-being because of the challenges they have faced. They might underreport their difficulties for social desirability, e.g. wanting to give positive feedback to the team, and even for fear of poorer social integration. The familiar relationship between our nurses and children might also influence their response to please their personal medical caregiver.

There are some weaknesses of our study: We didn’t carry out the same survey on a healthy, i.e. non-transplanted, control group. Still, we dare to assume that the answers from healthy children would have been comparable to the ones we obtained from our transplanted children and their parents, since almost all of those assume to do well – which we can suppose do healthy children, too. Further, we didn’t analyze our children’s HRQOL using a standardized questionnaire, as reported in the cited studies [6,7], which would have given us an “unbiased” control of the “real” HRQOL. Yet, even without these control groups this succinct clinical, observational study allows conclusions: The literature is clear and unequivocal, that is children after LT have a lower HRQOL [6-9]. This awareness is challenged after analysis of our questionnaire: our children and their parents believe that they do wonderfully well, even though the questions used in the interview were the same as asked in the reported literature; the only factor being different, was the fact that a personal, well-known nurse was present. The evidence based knowledge of the existing literature is an acceptable basis to allow us to conclude that our children and their parents were emotionally influenced during the interview. Nevertheless, further studies must be undertaken to demonstrate clear, valid evidence of our findings.

## Conclusion

This succinct data suggests that the perception of the patient’s well being, when inquired by the medical staff at the annual follow-up visit, seems incorrect. This study emphasizes the importance of routine standardized diagnostic procedures regarding cognitive and psycho-social development before and after transplantation in these children, as well as the importance of a professional mental health provider familiar with transplant issues being a necessary member of any transplant team in order to evaluate the real psycho-social outcomes of our liver transplanted children.

## Abbreviations

LT: liver transplantation; HRQOL: health related quality of life.

## Competing interests

The authors declare that they have no competing interests.

## Authors’ contributions

AC: Has made substantial contributions to the conception and design of the study, to the acquisition of data, and the analysis and interpretation of data; has been involved in drafting the manuscript. VMcL: Has been involved in revising the manuscript critically for important intellectual content. DB: Has been involved in revising the manuscript critically for important intellectual content. BEW: Has made substantial contributions to the conception and design of the study, and to the analysis and interpretation of data; has been involved in drafting the manuscript and revising it critically for important intellectual content; and has given final approval of the version to be published. All authors read and approved the final manuscript.
